# Therapeutic alliance in guided internet-delivered cognitive behavioural therapy: a thematic analysis of patients with non-cardiac chest pain

**DOI:** 10.1186/s40359-026-05179-w

**Published:** 2026-07-15

**Authors:** Nils Hedman Äng, Josefin Särnholm, Gerhard Andersson, Peter Johansson, Ghassan Mourad

**Affiliations:** 1 Department of Health, Medicine and Caring Sciences, Linköping University, Linköping, Sweden; 2 Department of Clinical Neuroscience, Karolinska Institute, Stockholm, Sweden; 3 Department of Behavioural Sciences and Learning, Linköping University, Linköping, Sweden; 4 Department of Health, Education and Technology, Luleå University of Technology, Luleå, Sweden; 5 HEI-Lab: Digital Human-Environment Interaction Labs, Lusófona University, Lisboa, Portugal; 6 Department of Internal Medicine in Norrköping, Vrinnevi Hospital, Norrköping, Sweden

**Keywords:** Internet-delivered cognitive behavioural therapy, Therapeutic alliance, Non-cardiac chest pain, Cardiac anxiety, Qualitative research, Thematic analysis, Digital mental health

## Abstract

**Background:**

Therapeutic alliance is an important aspect of psychotherapy and has been associated with treatment outcomes, but less is known about how it is experienced and maintained in guided internet-delivered cognitive behavioural therapy (iCBT), particularly when treatment concerns persistent physical symptoms such as non-cardiac chest pain (NCCP). This study explored how participants in an 8-week guided iCBT programme for NCCP experienced the therapeutic alliance, and how their accounts related to established alliance models.

**Methods:**

This qualitative interview study was conducted alongside a randomised controlled trial evaluating an eight-week guided iCBT programme for adults with NCCP and cardiac anxiety. Semi-structured interviews were conducted with 22 participants who had completed the programme. The interviews were audio-recorded, transcribed verbatim, and analysed using reflexive thematic analysis informed by Bordin’s model of therapeutic alliance, while remaining open to inductive theme development. Descriptive statistics were used to summarise participant characteristics.

**Results:**

The analysis generated three themes. First, agreement on goals and tasks was shaped by whether participants experienced the programme as personally relevant and whether treatment tasks could be balanced between fit and challenge. Second, the emotional bond between participant and therapist was supported by timely, attuned, and appropriately calibrated written feedback, although participants differed in their preferred level of emotional closeness and professional distance. Third, acceptability of the digital medium for collaboration encompassed the delivery format, the online platform, and the treatment content. Across themes, participants described alliance in the digital context as shaped by processes of negotiation in which therapist responsiveness helped them make sense of the treatment rationale, manage strain, and sustain engagement.

**Conclusions:**

In guided internet-delivered cognitive behavioural therapy for non-cardiac chest pain, therapeutic alliance appeared to depend not only on agreement about goals, tasks, and emotional bond, but also on the acceptability of the digital medium through which collaboration occurred. The findings suggest that therapists delivering text-based internet interventions may need to actively calibrate task demands, relational tone, and symptom explanations to support engagement and collaboration.

**Trial registration:**

This qualitative study was conducted within the registered parent trial: ClinicalTrials.gov NCT06136494; registered on November 13, 2023.

**Supplementary Information:**

The online version contains supplementary material available at 10.1186/s40359-026-05179-w.

## Introduction

Non-cardiac chest pain (NCCP) refers to recurrent episodes or persistent sensations of chest pain that resemble angina but occur in the absence of identifiable cardiac disease [1]. It is a common and costly condition, with prevalence estimates ranging from 10% to 30% in community samples and accounting for a substantial proportion of emergency department visits [2]. Despite the absence of cardiac pathology, NCCP is often perceived as threatening and is associated with persistent pain, functional limitations, and sustained psychological distress [3]. The condition is frequently associated with anxiety, depression, and cardiac-related fears, contributing to high healthcare utilization and reduced quality of life [2, 4]. A key mechanism is cardiac anxiety, where benign bodily sensations are misinterpreted as dangerous, leading to heart-focused attention, interoceptive fear and avoidance behaviour [5, 6].

Cognitive Behavioural Therapy (CBT) has been evaluated as a treatment of NCCP and found to have modest to moderate benefit according to a Cochrane review [7]. Nevertheless, patients with NCCP remain underrecognised and undertreated, often experiencing prolonged symptoms without adequate help, resulting in both individual burden and societal costs [4]. Access to traditional face-to-face CBT remains limited [8], prompting the development of scalable alternatives such as internet-delivered CBT (iCBT). To address this gap, our research group developed a guided iCBT-programme targeting the cognitive and behavioural processes associated with cardiac anxiety. The programme was evaluated within a registered randomised controlled trial, the outcomes of which have not yet been published. By helping participants reinterpret bodily sensations and re-engage in meaningful activities, the programme aimed to reduce fear and avoidance related to cardiac concerns.

Beyond clinical outcomes alone, it is essential to understand the non-specific therapeutic processes that make iCBT acceptable and effective. One such process is the therapeutic alliance, which has been shown to play a central role across psychological interventions [9].

Among existing models of the therapeutic alliance, Bordin’s [10, 11] framework remains the most commonly used [12]. It conceptualises the alliance as consisting of three interrelated components: agreement on therapeutic goals, agreement on the tasks required to achieve them, and the emotional bond between therapist and patient. Although numerous alternative conceptualizations have been proposed [13], most of them share Bordin’s core idea of the alliance as a collaborative relationship directed toward helping the patient achieve meaningful change [9]. Bordin’s model has also been complemented by later work emphasising the dynamic and sometimes strained nature of alliance formation. Safran and Muran [14], for example, draw attention to how therapeutic collaboration is shaped through the negotiation of expectations, tasks, relational needs, and tensions that may arise during therapy.

The therapeutic alliance has often been described as one of the most consistent factors in determining treatment outcome [9]. Yet many researchers and clinicians question whether a deep and authentic relationship can truly be established in a format without synchronous communication or nonverbal signals [15]. This raises important questions about the conditions that facilitate alliance in digital formats. Recent developments make such research especially timely: during the COVID-19 pandemic, digital formats became central to the provision of care [16], and in the emerging age of artificial intelligence there is growing attention to the role of mediated human contact in therapy [17].

Research has demonstrated that internet-delivered therapy can foster a strong therapeutic alliance, and alliance has also been shown to predict treatment outcome in this format, although findings across studies remain somewhat mixed [18]. In a systematic scoping review of text-based digital psychotherapy, van Lotringen et al. found generally high alliance ratings and mostly positive associations with treatment outcomes. However, not all studies reported significant associations, and the strength of the alliance–outcome relationship varied across studies and outcome measures [18]. Thus, current evidence suggests that alliance is relevant in internet-delivered therapy, while its role may depend on treatment format, measurement approach, and clinical context.

However, the frameworks that have guided most measures and theoretical discussions on the therapeutic alliance were originally developed for face-to-face therapy [18, 19]. When applied to digital contexts, the alliance has often been treated as a direct extension of the same principles [20]. Yet, as Saxler et al. note, this assumption may obscure processes that unfold differently in online contexts, particularly around how misattunement emerges and is addressed [13]. Additional factors that have been suggested as relevant for alliance in internet-based interventions include empathy [18], availability, interactivity [21], user engagement, facilitators and barriers [17], and programme usability [22]. Despite these contributions, digital alliance is still largely understood through top-down extensions of face-to-face models rather than through frameworks grounded in the lived experience of online therapy [13]. This underscores the need for qualitative research exploring how participants themselves understand and negotiate alliance in internet-delivered interventions.

To date, we have only been able to identify two studies [23, 24] that used qualitative interviews with participants to construct a specifically digital conceptualization of the therapeutic alliance in internet-delivered interventions. Both combined Bordin’s model as a guiding framework with openness to data-driven refinements. Both reported goals and tasks in close connection to each other in their thematic structures, with goals portrayed as subordinate to or embedded within tasks rather than as a fully separate category. In addition, both extended Bordin’s model by adding *usability heuristics* as a fourth component, reflecting participants’ experiences of how digital features promote engagement, self-discovery, and autonomous problem-solving. Barceló-Soler et al. further introduced *anonymity* as a new subtheme, reflecting the importance participants placed on privacy in the face of stigma around mental illness [23]. Taken together, these findings suggest that while the core components of Bordin’s model remain relevant, the therapeutic alliance in internet-delivered interventions also involves unique dimensions that are not fully captured in traditional frameworks.

In addition to the previously discussed complexities of conceptualizing digital alliance, little is known about how such alliance processes are experienced in interventions for persistent physical symptoms, including persistent non-cardiac chest pain. Persistent physical symptoms are increasingly understood through biopsychosocial and symptom perception models, in which symptom experience is shaped by interactions between bodily input, prior experience, expectations, attention, emotions, behaviour, and healthcare encounters [25]. In this context, treatment often requires more than symptom reduction alone. It also involves helping patients develop explanations that validate bodily experience without reducing symptoms to either purely biomedical pathology or purely psychological causes [26]. This may be particularly relevant for the therapeutic alliance, since agreement on goals and tasks may depend on whether the treatment rationale feels credible, respectful, and applicable to the patient’s own symptom experience.

NCCP can be understood as one example of this broader clinical challenge. Although patients have been medically assessed for cardiac disease, their symptoms are experienced as bodily, potentially threatening, and often difficult to interpret. Guided iCBT for NCCP therefore asks participants to engage with a biopsychosocial rationale and with tasks targeting cardiac anxiety, symptom-focused attention, avoidance, and re-engagement in valued activities. While similar attributional processes are also relevant in other iCBT contexts, NCCP offers a clinically important setting in which to examine qualitatively how participants experience the therapeutic alliance when psychological and behavioural treatment is applied to persistent and threatening bodily symptoms.

Taken together, this highlights the value of qualitative research that can clarify how therapeutic alliance is formed and maintained in digital interventions for conditions such as persistent non-cardiac chest pain, where distressing bodily symptoms remain central to the treatment process. Although existing research shows that internet-delivered therapy can support therapeutic alliance, less is known about how participants experience alliance when psychological and behavioural treatment is applied to persistent and threatening bodily symptoms. This study therefore aims to explore how participants who remained engaged in an iCBT programme for NCCP experienced the therapeutic alliance, including how it was formed and maintained within a primarily text-based digital treatment context, to identify perceived supportive and challenging features, and how participant accounts align with or challenge established theoretical models.

## Methods

### Design

This qualitative interview study employed a thematic analysis and was conducted alongside a randomised controlled trial (RCT) evaluating iCBT for participants with NCCP and cardiac anxiety. The RCT included two arms: an intervention group who received an 8-week guided iCBT programme, and an attention control group who received weekly non-specific contact and were offered iCBT after three months. A total of 134 participants were randomised using a 1:1 allocation ratio. Reporting of the qualitative data adheres to the COREQ guidelines (Consolidated Criteria for Reporting Qualitative Research) [27]. As the present study was embedded within a randomised controlled trial, trial-related aspects are reported in accordance with the CONSORT 2025 [28] guidelines where applicable. A completed CONSORT checklist is provided as Additional file 1.

### Intervention

The iCBT programme consisted of eight structured modules delivered over eight weeks via a secure online platform (iterapi.se) [29]. Modules covered psychoeducation, goal setting, exposure to physical activity and bodily sensations, mindfulness, and acceptance strategies. The programme aimed to support cognitive change across components by helping participants reframe and reinterpret bodily symptoms. Participants were expected to complete one module per week and to carry out related therapy tasks. Each participant received weekly written feedback and support from the same therapist, with therapist time averaging around ten minutes per week. All therapeutic contact was text-based. Telephone contact was used only when needed to resolve technical difficulties. The programme could be accessed via computer or mobile device using secure login (national electronic ID or SMS-based two-factor authentication).

### Participants and recruitment

For the present qualitative study, 22 participants were recruited exclusively from participants who had engaged in the guided iCBT programme throughout the treatment period (See Table 1).

**Table 1:** Participant characteristics

Participant characteristics (*N* = 22)	Frequency	%
Male	9	40.9
Female	13	59.1
Age, years (Mean ± SD)	49 ± 19.3	
Marital status		
Married or living with partner	13	59.1
Single / Not living with a partner	8	36
Widowed	1	4.5
Educational level		
University/College	10	45.5
Vocational school/Upper secondary school	8	36.4
Primary school	2	9.1
Other	2	9.1
Occupational status		
Employed	15	68.1
Retired	5	22.7
Student	2	9.1
Country/region of origin		
Sweden	20	90.9
Nordic country	1	4.5
Asian country	1	4.5
Interview duration mins (Mean ± SD)	36.3 ± 12.3	

The interviews were conducted from June to December 2024 within the framework of the IKSIT trial, a Swedish acronym derived from Icke-Kardiell bröstSmärta: Internetförmedlad Terapi [Non-Cardiac Chest Pain: Internet-Delivered Therapy], at Linköping University, Sweden, with participants recruited nationwide via online advertisements. The RCT included 67 participants in each arm. Participants were adults with recurring or persistent chest pain without an identified cardiac cause following appropriate medical evaluation, together with elevated cardiac anxiety. In the intervention arm, 8 of 67 participants did not complete the post-treatment assessment, corresponding to a post-treatment assessment attrition rate of 11.9%. Treatment engagement, measured as completed modules, had a median of 7 modules (IQR = 3) among all participants allocated to the intervention, compared with 8 modules (IQR = 1) among participants included in the present interview study. Nineteen interview study participants came from the intervention arm, and three from the control arm, who later received iCBT after three months. Recruitment took place continuously as participants concluded the treatment period. Following the post-measurement, they were contacted by email and invited to take part in interviews. Sample adequacy was considered in terms of information power [30]. The sample was judged to provide sufficient information power because the study aim was focused, participants had direct experience of the guided iCBT programme, the analysis was informed by established alliance theory, and the interviews provided rich accounts relevant to the research question.

To minimise bias, none of the participants had any prior therapeutic relationship with the interviewer. In a few cases, the interviewer had previously conducted the inclusion interview, but this did not involve therapeutic contact or ongoing clinical involvement. All participants provided written informed consent prior to the interview.

### Data collection

Data were collected through semi-structured interviews conducted via video conference (Zoom). Only audio was recorded, using a digital voice recorder, and the recordings were transcribed verbatim. Interviews were conducted by three trained clinicians: a female registered nurse with a PhD, the first author (NHÄ), a male licensed clinical psychologist and PhD student, and the last author (GM), a male registered nurse and associate professor.

The interview guide (see additional file 2) was theory-driven, informed by Bordin’s model of therapeutic alliance [11], as well as previous research on digital alliance, including findings related to usability reported by Doukani et al. [24] and Barceló-Soler et al. [23]. It consisted of open-ended questions with optional prompts intended to elicit participants’ experiences of collaboration and emotional bond, including how treatment tasks and goals were perceived as aligned and how participants experienced being understood, supported, and accompanied within the therapeutic relationship. The majority of interviews were transcribed manually by the first author as part of the familiarisation process. For a few interviews, an offline transcription tool installed on the first author’s computer was used to generate a preliminary transcript. These transcripts were subsequently checked and corrected manually against the audio recordings. No external transcription service was used.

### Data analysis

The data were analysed using reflexive thematic analysis as described by Braun and Clarke [31, 32], supported by NVivo software for data organisation and coding. The analysis was best understood as a theory-informed reflexive thematic analysis. Bordin’s model of the therapeutic alliance was used as a theoretically informed point of orientation rather than as a fixed coding frame. Thus, the concepts of goals, tasks, and emotional bond informed the early stages of coding, while the analytic process remained open to meanings and patterns that extended, complicated, or did not fit these components. The analysis involved an iterative movement between existing theory and participants’ accounts.

The first author began by reading the transcripts repeatedly to become familiar with the dataset and to note initial impressions related to participants’ experiences of collaboration, therapist contact, treatment tasks, treatment goals, and the digital format. Initial coding focused on segments of text describing how participants experienced the therapeutic relationship, made sense of the treatment rationale, engaged with or struggled with treatment tasks, and experienced the digital treatment context. Some codes were informed by Bordin’s model and previous literature on digital alliance, whereas other codes were developed more openly from the data. This was particularly important when participants described aspects of the treatment medium, programme content, platform, or delivery format that shaped collaboration but were not readily captured by the traditional alliance components.

Coding and theme development attended to both semantic and latent meanings. Semantic meanings included participants’ explicit descriptions of what they found helpful, challenging, supportive, frustrating, trustworthy, or difficult to engage with. Latent meanings were explored by considering the underlying values, assumptions, and tensions expressed in these accounts. For example, differing preferences regarding therapist feedback were interpreted not only as individual opinions about communication style, but also as reflecting a broader tension between emotional closeness and professional distance. Similarly, accounts of weekly modules and treatment tasks were interpreted in relation to tensions between structure and flexibility, challenge and support, and autonomy and guidance.

Codes were iteratively organised into candidate patterns related both to Bordin’s alliance components and to emerging digital-specific processes. During this process, the initial intention to categorise factors as either promoting or hindering alliance was replaced by a more interpretive focus on how participants negotiated contrasting needs and preferences within the treatment context. Candidate themes were reviewed in relation to the full dataset and refined for internal coherence, conceptual clarity, and relevance to the research aim. This involved moving back and forth between coded extracts, whole transcripts, developing themes, and existing alliance theory.

Several members of the research team contributed to the analytic process by independently reading subsets of transcripts and discussing developing codes, candidate themes, and alternative interpretations. These discussions functioned as reflexive analytic dialogue, helping the first author consider how different clinical and disciplinary perspectives might shape the interpretation of the material. Alternative readings were discussed in relation to the research question, the full dataset, and the developing thematic structure. Final theme construction remained the responsibility of the first author, in dialogue with the co-authors.

Themes were then refined, named, and organised into a thematic structure that captured both continuity with established alliance theory and aspects of alliance that appeared specific to the digital treatment context. Illustrative quotations were selected to represent central patterns in the data while also preserving variation between participants. A thematic map was created to depict relationships between themes and subthemes.

### Reflexivity and analytic stance

The analysis focused on participants’ accounts as situated interpretations of alliance-related experiences within a specific treatment context. We did not treat the therapeutic alliance as a fixed entity to be directly measured, but as a clinically meaningful construct shaped by participants’ experiences, the treatment rationale, the digital context, and the interaction with the therapist. The purpose of the analysis was therefore not to determine the strength of the alliance, but to interpret how participants made sense of alliance-related processes in guided internet-delivered treatment.

The research team brought different professional and disciplinary perspectives to the analysis, including clinical psychology, nursing, internet-delivered treatment, and cardiovascular care. These perspectives shaped what was noticed in the data and how emerging themes were interpreted. The first author is a licensed psychologist and PhD student with primary responsibility for the treatment programme and was therefore closely familiar with the intervention rationale, therapeutic content, and clinical aims. This familiarity supported a nuanced understanding of participants’ accounts, but also created a risk of interpreting the data in ways that confirmed the intended logic of the programme. To address this, analytic discussions explicitly considered alternative interpretations, tensions in the material, and ways in which participants’ accounts challenged the initial assumptions of the programme and of Bordin’s model.

The interviewers had different relationships to the broader study context. Some had been involved in inclusion interviews or in the treatment programme, although no interviewer conducted interviews with participants for whom they had served as therapist. Participants were informed about the study context and the interviewer’s role. Because positive accounts may have been shaped by social desirability, gratitude toward the treatment team, or the fact that participants had completed or remained engaged with the programme, the analysis attended not only to explicit praise but also to hesitations, qualifications, descriptions of strain, and indirect criticism.

## Results

The analysis generated three themes that together describe how therapeutic alliance was formed and maintained in guided iCBT for non-cardiac chest pain. Two of these themes, Agreement on goals and tasks and Emotional bond between participant and therapist, were primarily informed by Bordin’s model of therapeutic alliance and reflect a theory-driven analytic focus. Consistent with participants’ accounts, agreement on goals and tasks was treated as a single theme, as these components were experienced as closely intertwined rather than as distinct aspects of the alliance. A third theme, Acceptability of the medium for collaboration, was generated through a more inductive part of the analysis, as participants’ accounts drew attention to how features of the digital context shaped their ability to engage in and sustain a collaborative relationship. Whereas previous qualitative studies of digital alliance [23, 24] have treated usability heuristics as a relatively circumscribed dimension, participants’ accounts in the present study pointed to a broader set of conditions that needed to be acceptable for collaboration to be sustained. This theme therefore extends earlier conceptualisations by encompassing not only the technical usability of the treatment platform, but also the relatability of the textual content and the acceptability of the delivery modality itself.

**Fig. 1:**
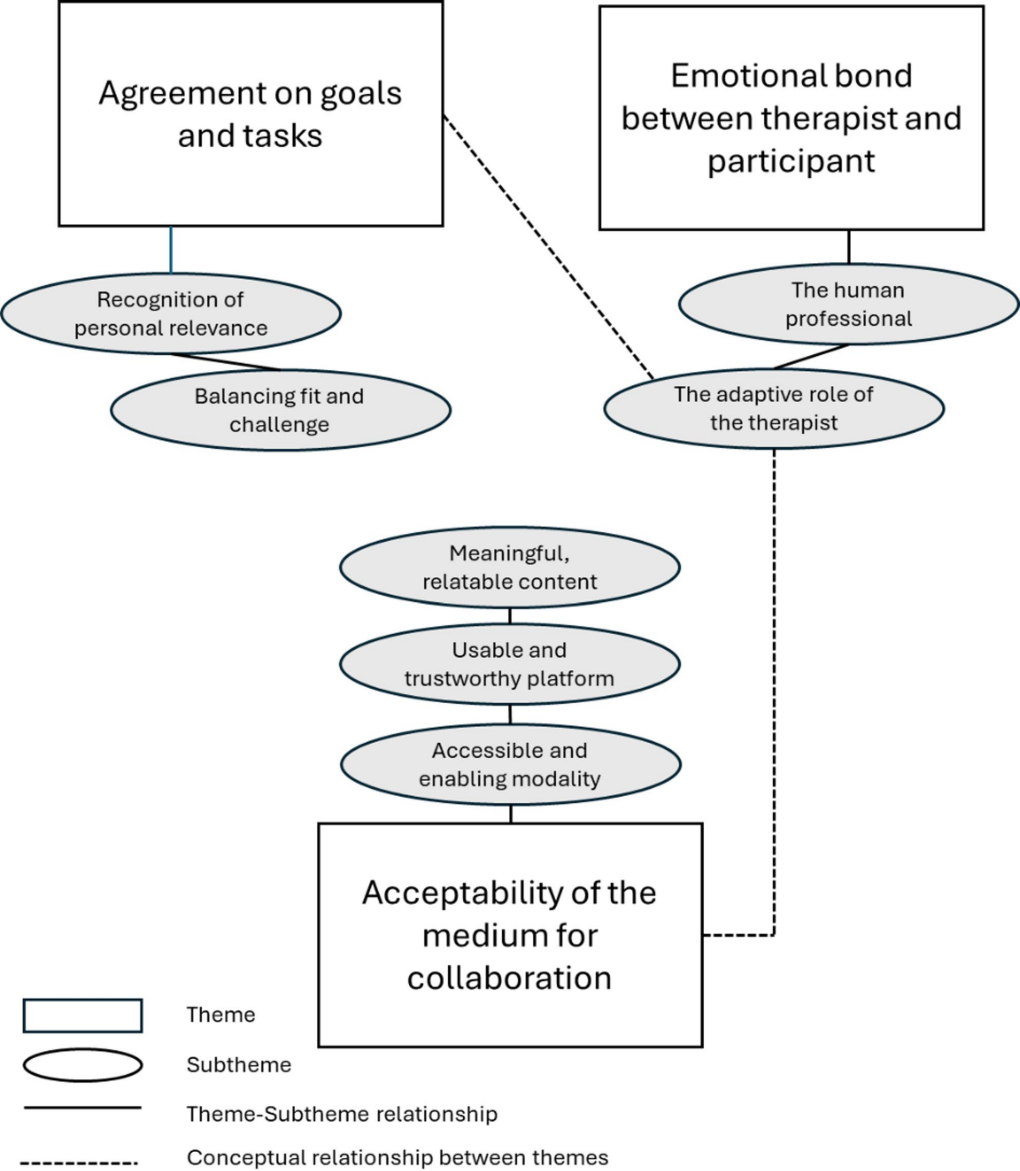
Thematic map of the therapeutic alliance in guided internet-delivered cognitive behavioural therapy for non-cardiac chest pain. The figure illustrates three themes and their associated subthemes. Solid lines indicate relationships between themes and subthemes, while dashed lines indicate conceptual links between themes. The subtheme “The adaptive role of the therapist” is intersected by these conceptual links to illustrate its function in linking the overarching themes by integrating participants’ experiences across alliance components

Each theme comprised two to three subthemes with seven subthemes in total. Figure 1 illustrates the relationships between themes and subthemes and highlights how the themes were conceptually linked.

Overall, participants described many aspects of the alliance as helpful, while also portraying it as a dynamic process shaped by diverse needs, preferences, and moments of strain. The therapist played a key role in navigating this process by adapting to participants’ expectations, addressing misalignments when these occurred, and helping them make sense of the treatment content and digital format.

### Agreement on goals and tasks

Participants’ accounts showed that agreement on goals and tasks was not a static condition but an evolving process that unfolded throughout treatment. What made the work feel meaningful depended on two intertwined dynamics: first, how participants came to view the treatment as personally relevant to their own symptom experience and life situation, and second, how they navigated the ongoing adjustments required to keep the tasks workable without losing their therapeutic value. These dynamics are illustrated in the two subthemes below.

### Recognition of personal relevance

When encountering the advertisement for the study, participants expressed varied initial reactions, ranging from enthusiasm and a sense of finally having found what they had been longing for to a more habitual resignation and disbelief that anything could help them. Nevertheless, accounts converged around entering the programme with at least an experimental mindset.

Participants described entering the programme with low expectations but a willingness to “give it a chance.” One participant explained, “I had no expectations… we’ll see if it works” (P3), while another stated, “I was a bit sceptical, but I thought I’d give it a chance” (P4), yet another: “I was open for any support I could get” (P9). Another participant framed her participation as simply “clicking in” and “seeing what happens”, when encountering the advertisement (P12).

Participants stressed the importance of a treatment programme that felt relevant to them personally, often in contrast to prior healthcare encounters where their concerns had felt dismissed or misunderstood.

When I joined this programme and had my first interview, it felt like the first time someone really listened to how I was actually feeling. At the health centre they only did an ECG, listened to my heart, and said everything was normal. There were no follow-up questions about other kinds of help. This programme made me realise that it’s completely okay to feel the way I do. (P5)

One of the most salient benefits described by participants was the informative nature of the initial educational sections of the programme. These early parts helped them make sense of their often-confusing symptoms and contributed to a sense of being understood rather than isolated.

It felt reassuring that my own problems were reflected in the examples. […] It helps the therapy process forward in a way to feel represented. (P3)

Participants described how this recognition sometimes opened the way for new insights and behaviour changes. Other participants had anticipated a more rapid and complete symptom relief but were encouraged by the notion that the treatment programme was a continuous process and that they could continue to use the tools they had learned after the study period was over.

### Balancing fit and challenge

After recognizing the value of the programme and engaging with its rationale, participants described an ongoing negotiation between how well the content suited their personal situation and how demanding it felt. This balance between *fit* and *challenge* was central to sustaining motivation and trust in the treatment. When the modules felt relevant, participants were more willing to engage with difficult tasks; when the demands felt excessive or the content seemed less applicable, the therapist’s role in adapting and re-framing the material became crucial.

[The therapist] told me not to see it as a failure, that I didn’t understand the task. It felt good to hear, that ‘it’s okay’. I don’t like feeling like I’ve failed something. (P5)

Participants differed in how they experienced the therapy tasks. For some, the tasks felt unusually tailored to their specific symptoms, whereas others experienced them as addressing more general aspects of mental health. The generality or specificity was not considered problematic in itself, although certain accounts described moments where parts of the programme felt like a poor fit. In such cases, the therapist was able to address the misalignment by accommodating the tasks and helping participants make sense of them.

[The therapist] was keen to adjust the content to my personal needs, which I appreciated. Above all the physical activity. My problem was too much physical activity, and he gave me other ways to think about [the module]. (P15)

Another recurring source of strain was the sense of demands and time pressure inherent in the tasks, as one participant stated that: “a week is a bit tight… it was stressful to keep up with the practical tasks” (P13). While some participants experienced challenge as motivating, arguing that they wouldn’t have achieved their goals without a therapist who both “supported and pushed” (P6), others highlighted the difficulty of fitting the tasks into daily routines and appreciated when accommodations were possible. Across accounts, being supported and guided throughout the tasks was described as highly important.

Experiencing positive effects from the tasks also played a crucial role in maintaining engagement. Early signs of reduced worry or increased understanding were described as reinforcing participants’ trust in the process and strengthening their motivation to continue with difficult or demanding exercises.

I feel that at certain stages it has given me good support and reduced my worry… above all, it has given me a belief that if I continue with this treatment, it will be helpful for me. (P10)

These moments of perceived progress functioned as confirmation that the effort invested in the tasks was worthwhile, helping participants persist even when the work felt challenging.

### Emotional bond between participant and therapist

Participants’ accounts highlighted that the emotional bond in iCBT was shaped by a combination of human connection and the constraints of the digital format. While the relationship unfolded through written communication, it carried many of the same relational tasks as face-to-face therapy: conveying understanding, responding to distress, and providing a sense of being accompanied throughout the treatment. At the same time, the absence of physical presence brought participants’ preferences and sensitivities into sharper relief, resulting in a wide range of desired levels of closeness, professionalism, and emotional depth. Despite this variability, the bond was sustained when the therapist responded in ways that felt attuned, genuine, and appropriately calibrated to the participant’s needs. These dynamics are captured in the two subthemes below.

#### Adaptive role of the therapist

Faced with differing expectations, needs, and preferences, the therapist had a bridging and supportive role in explaining, guiding, and accommodating participants throughout the therapy tasks. This role also involved helping participants make sense of the treatment content, attending to relational and support needs, and facilitating use of the digital platform.

The therapist also had an important role in responding to participants. Participants consistently emphasised the value of responses that were both quick and informative, as these reinforced their sense of agency and relationship, reminding them that there was someone on the other side of the screen and that their actions mattered.

With quick feedback, you could see that [the therapist] had read what you wrote. And it wasn’t an automatic response. (P11)

On a deeper level, the therapist’s responsiveness was expressed through matching the participants’ emotional tone, response length, and choice of language. Participants noted that they felt more understood when the therapist’s language mirrored their own.

It was like [the therapist] used my language so to speak. Like he wrote in the same way as me and used the same words. He helped the thoughts move along, if I wrote a reflection, he wrote something that helped me reflect a step further. (P19)

#### The human professional

Participants described strikingly varied preferences regarding the emotional depth of their relationship with the therapist. Some valued a more distanced and professional stance, emphasizing clarity and seriousness in communication.

The responses were professional, the way you would expect from a staff member to another person. Serious. Not emojis or thumbs up or anything like that, but proper, professional responses like from healthcare staff. (P18)

Others preferred warmth, authenticity, and empathy, appreciating when the therapist conveyed genuine human connection:

He wrote in a very kind and understanding way. I felt seen. It was also comforting that, beyond the content which you know is just a tool or a text, the personal or human contact still felt very genuine to me. (P14)

Preferences also varied in relation to emotional intimacy. Some participants described that pictures, videos, or the possibility of a video call could have made the therapist feel more personally present, whereas others found such features unnecessary or even valued the relative distance of the text-based format. Comparisons with face-to-face therapy also reflected these differences, as in-person contact can sometimes feel uncomfortable:

He would be able to read me in a different way, my facial expression, my eyes, I would feel more exposed. (P18)

Others, on the other hand expressed an expectation that face-to-face therapy would allow conversation to flow more naturally and deeply.

Despite these differing preferences, participants consistently portrayed the therapist as both human and professional. Someone who could balance warmth with structure and attune to each participant’s relational needs. The ability to flexibly adjust communication style and level of emotional engagement emerged as essential for maintaining trust and supporting participants’ sense of safety within the digital format.

### Acceptability of the digital medium for collaboration

In order for the collaborative relationship to work, the digital medium that makes the relationship possible must at least be acceptable. In our analysis, this medium that needed to be acceptable consisted of three parts: the delivery modality itself, the online platform, and the content of the modules which consisted of texts, video, images and audio.

#### Accessible and enabling modality

Most participants did not compare internet-delivered treatment primarily to face-to-face treatment, but to their usual care, which was sporadic contact with medical personnel, often with new physicians each time. Against that backdrop, the online format was frequently experienced as offering a more reliable and continuous connection than they were accustomed to.

Previously, it had always been: “Oh you don’t feel so well… Why could that be? Go home and do this.” But never before that “you are not alone in this.” (P1).

I think it’s good that you have ONE person that you know is your [therapist] and that you can turn to. It’s like when you go to the doctor, you prefer to meet the same person every time. (P2)

Those with experience of face-to-face treatment described iCBT as more flexible regarding time and place.

It feels like I’m my own boss. I decide how much I do and when I do it. And I like it. (P15)

However, participants noted a lack of flexibility regarding the tasks themselves, which were presented as a predefined package rather than collaboratively negotiated.

I was tired and had work and stuff. But now I have to go out and do my exposure therapy. Because that’s what it says. (P13)

Adaptations could nevertheless be made through dialogue with the therapist, who helped participants interpret and apply the tasks in ways that felt personally relevant.

[The therapist] could probably tell that this didn’t suit me. That there were other areas that I find more important. (P7)

I thought “I can do it my way and [the therapist] will give his impression of what it is I’ve done”. And it went really well, I received much encouragement and praise. (P14)

Across accounts, participants described a workable balance between flexibility and enough structure to support progress.

#### Usable and trustworthy platform

A well-functioning and user-friendly platform was considered important, since low usability could become a barrier to completing therapy tasks, particularly for participants already pressured in their daily routines.

It worked smoothly. There were different chapters so you remember where you were before when you started reading at work. And then you come home you could continue. (P13)

The [login function] itself wasn’t difficult to use, but it felt rather cumbersome. That made me postpone doing it. (P2)

The platform also needed to convey a sense of professionalism. For some, trust was reinforced by the university affiliation and the visible presentation of the study team.

It’s important that you could see the whole team. It’s important to know that it’s not some anonymous call centre but you know the persons involved. Otherwise, you won’t know if there’s a new person each time. (P10)

The secure login features provided a sense of safety and anonymity. Some participants described this partial anonymity as helpful, making it easier to confide, whereas others did not regard anonymity as particularly important.

#### Meaningful and relatable content

The textual and video content of the treatment modules included psychoeducation, rationales, instructions and clinical examples to help participants make sense of them.

Participants emphasised that the content needed to be informative, accessible, meaningful, and relatable. Relatable examples helped participants feel validated in their experiences.

I thought it was so interesting to hear from other patients. See how it was for them. There was so much I could relate to and it was good to not feel alone. (P11)

Even when participants’ symptoms differed from those described in the vignettes, they described being able to adapt the material to their own situation.

For me the big thing was high pulse rather than chest pain, but I found that the examples worked at least as well for the difficulties I experienced. (P8)

Participants also expressed a preference for content that was novel and introduced new ideas, but it needed to remain sufficiently attuned to their existing understanding of symptoms in order to be meaningful. Across accounts, the psychological focus of the treatment was presented as understandable and acceptable, even among those who initially emphasised somatic explanations.

## Discussion

The aim of this study was to explore how participants in an iCBT programme for NCCP experienced the therapeutic alliance. The analysis generated three overarching themes: agreement on goals and tasks, emotional bond between participant and therapist, and acceptability of the digital medium for collaboration. Taken together, these themes suggest that, among participants in this study, negotiated and dynamic processes contributed to the maintenance of alliance in iCBT for NCCP. Within these processes, therapists can be understood as helping to balance diverse and sometimes conflicting needs related to goals, tasks, relational expectations, and the digital context. Helping participants balance diverse and sometimes conflicting needs in a digital context.

### Agreement on goals and tasks

For participants in this study, agreement on goals and tasks was closely tied to whether the intervention felt personally meaningful and useful. Many entered the programme with a mix of resignation and cautious hope after previous healthcare encounters where their symptoms had been dismissed or left unexplained. In line with earlier qualitative work on iCBT for NCCP, participants described the programme as trustworthy and helpful when it offered an opportunity to understand their chest pain and regain a sense of normal life [33].

Participants rarely talked about “task agreement” in isolation. When they described being willing to engage in demanding exposure or behaviour change exercises, this was almost always tied to a sense that the tasks had already proven their value or had the potential to do so. This is why “recognition of personal relevance” emerged as a central subtheme: agreement about tasks was inseparable from experienced or anticipated benefits. This pattern supports reciprocal models of alliance, in which alliance and outcome are mutually reinforcing rather than unidirectional [34].

The context of persistent physical symptoms further shaped how goals and tasks were negotiated. By the time they enrolled in this explicitly psychological intervention, most participants had accepted that behavioural and emotional factors contributed to their symptoms, yet many still found this connection confusing. All accepted the overall rationale for iCBT, but some remained uncertain about how it applied to their own situation. This echoes findings from other studies of persistent somatic symptoms, where acceptance of a biopsychosocial explanation is gradual and often conditional [35]. Participants in the present study highlighted the importance of tasks that were credible and sufficiently tailored to their symptom experience, and they valued therapists who could accommodate mismatches by reframing exercises or adjusting expectations without abandoning the rationale. This *scaffolded learning* [36] around symptom attribution suggests that the task component of alliance in iCBT for NCCP cannot be reduced to compliance. It requires ongoing pedagogical work to maintain a workable fit between the treatment model and lived experience.

### Emotional bond between participant and therapist

Participants’ descriptions of the emotional bond with their therapist closely resembled those reported in previous qualitative studies of iCBT for NCCP [33] and for depression among cardiovascular patients [37]. In these studies, patients described the programme as “safe”, “trustworthy” and “supportive”, and emphasised that the therapist’s feedback made them feel taken seriously and no longer alone with their problems. Participants in the present study similarly stressed the importance of feeling seen and understood through written feedback. They valued responses that were timely, informative, and formulated in a language that mirrored their own, and they described this as a sign that the therapist was genuinely engaged with their situation.

At the same time, preferences for emotional closeness varied considerably. Some participants preferred a more formal and professional tone whereas others appreciated warmth, encouragement, and signs of the therapist’s personality. Rather than indicating inconsistency, these differences point to the therapist’s role in flexibly regulating the level of intimacy and immediacy to match individual needs.

These findings resonate with Safran and Muran’s [38] conceptualisation of the therapeutic alliance as shaped by a continuously negotiated relationship in which tensions and misunderstandings may arise. In our material, such tensions rarely took the form of explicit interpersonal conflict. Instead, participants described strain in relation to demanding tasks, perceived misalignment with the programme content, or uncertainty about whether they had “failed” an exercise. In these situations, therapist responses were described as helpful when they normalised difficulties, validated participants’ efforts, and collaboratively re-framed exercises. In this sense, the emotional bond in iCBT for NCCP may be understood not simply as a by-product of successful task completion, but as a resource for helping participants navigate moments when the structure of the programme felt difficult to reconcile with their needs, expectations, or vulnerabilities.

### Acceptability of the digital medium for collaboration

Our findings also speak to ongoing debates about whether usability heuristics should be considered a separate dimension of the digital therapeutic alliance. Previous qualitative work in iCBT has proposed adding usability heuristics alongside goals, tasks, and bond, based on participants’ reports of how design features facilitated engagement [23, 24]. Our analysis also resonates with Zalaznik et al.’s [39] distinction between alliance with the programme and alliance with the therapist. In their study of iCBT for panic disorder, patients’ alliance with the programme predicted subsequent symptom reduction, whereas alliance with the therapist predicted adherence [39]. This distinction supports the view that the programme and the digital medium are not merely neutral channels for therapist contact but may form part of how collaboration is experienced and sustained in internet-delivered treatment. In the present study, participants did not always discuss the technical platform in detail, but platform-related issues became clinically meaningful when they affected ease of use, trust, or engagement. Thus, complaints about login procedures or other technical features should not be understood as contradicting the broader finding, but rather as examples of how the medium becomes more visible when it creates friction in the therapeutic process. Based on these accounts, we suggest broadening the notion of usability into the more encompassing concept of *acceptability of the medium for collaboration*. This includes the technical platform, the delivery modality, and the textual and audiovisual content of the modules. Participants appreciated the flexibility to complete tasks at a time and place of their own choosing and contrasted the continuity of having “one therapist” with previous experiences of fragmented medical care. Similar to cardiovascular patients in Westas et al. [37], many described the programme as offering a kind of freedom and constant availability that differed from intermittent clinic visits.

At the same time, the digital medium imposed constraints. Tasks were perceived as a predefined package, and some participants experienced the weekly pacing and time-limited assignments as stressful. Participants described therapist responsiveness as important in helping them navigate such constraints, for example when therapists reframed ill-fitting exercises, clarified expectations, or helped participants find workable ways of approaching the tasks. Rather than indicating that the medium was either supportive or obstructive in itself, these accounts suggest that its acceptability depended on how the platform, content, delivery format, and therapist guidance worked together in practice. From an alliance perspective, the medium may therefore be understood as one of the conditions through which collaboration in iCBT is made possible, maintained, or strained. It may remain relatively unobtrusive when it supports the therapeutic process sufficiently, but become salient when it interferes with trust, accessibility, or perceived fit, such as when logins feel cumbersome or when content feels too generic. In this sense, therapist responsiveness appeared to help participants manage limitations of the medium.

### Processes of negotiation in the digital alliance

Across the themes, participants’ accounts suggested that alliance in guided iCBT was not experienced as a static quality of the therapeutic relationship, but as something shaped through ongoing adjustments between participant needs, therapist responsiveness, treatment tasks, and the constraints and affordances of the digital format. We use the term negotiated digital alliance to describe this interpretive synthesis. Participants depicted an ongoing balancing act between contrasting values: between a narrow or general focus, different levels of challenge in tasks, between autonomy and relatedness in the relational domain, and between flexibility and structure in the digital medium. Many accounts reflected a dialectical pull between the desire to “be one’s own boss” and the need for guidance, reassurance, and containment. These dynamics echo the idea that alliance is not static but a process that is continually co-constructed and re-constructed.

In digital contexts, Saxler et al. [13] have cautioned that conflicts may be particularly risky, as frustration with an app or web platform may simply lead to disengagement rather than resolution. Our findings suggest, however, that the presence of a human therapist may help buffer against such risks when therapists explicitly acknowledge the medium’s limitations, offer alternative ways of approaching tasks, and remain attentive to signs of strain. In that sense, the digital alliance in iCBT for NCCP may not be adequately understood as merely a translation of face-to-face constructs into an online format, but as a relationship that is negotiated within the possibilities and constraints of mediated communication.

### Methodological considerations

Several methodological aspects may influence the trustworthiness and transferability of the findings. The sample consisted of participants who remained reachable after the treatment period and who had completed or remained highly engaged with the guided iCBT programme. This sampling frame strengthened the study’s ability to generate rich accounts of how alliance could be formed and maintained during treatment, including how participants described negotiating strain, task demands, symptom-related uncertainty, and the limitations of a primarily text-based digital format. However, it also limits the conclusions that can be drawn.

Participants who disengaged earlier, experienced more severe misalignment with the programme, felt insufficiently supported by the therapist, or found the digital format unacceptable may be underrepresented. The findings may therefore privilege accounts of relatively successful alliance formation and should not be interpreted as representing the full range of alliance experiences in the intervention arm. In particular, the study cannot determine how alliance processes unfolded among participants whose difficulties were not resolved or who discontinued treatment before the end of the programme. Future research should specifically examine alliance processes among participants with lower adherence, weaker treatment fit, or negative experiences of digital care, since these accounts may reveal different forms of strain, disengagement, or unmet support needs.

In line with a theory-focused understanding of qualitative generalisation [40], the purpose of the study was not statistical representativeness but conceptual refinement. The themes should therefore be understood as a contextually situated analysis of how alliance was experienced and constructed among engaged participants in a guided iCBT programme for NCCP. Rather than being generalisable to a population in a sampling-based sense, the findings may contribute to broader theoretical understanding of how alliance processes are shaped by task demands, therapist responsiveness, symptom attribution, and the constraints of primarily text-based digital care. The detailed description of the intervention, sampling frame, and participant characteristics is intended to support readers in judging the transferability of the findings, while the main contribution of the study is theory-focused: to refine understanding of how therapeutic alliance may be constructed and maintained in primarily text-based digital treatment for persistent physical symptoms.

The use of three interviewers broadened perspectives but may also have introduced variability in interviewing style, which could affect the depth and direction of participants’ accounts. Several members of the research team engaged independently with parts of the material, and team-based analytic discussions were used to bring different disciplinary and clinical perspectives into the interpretation. This supported reflexivity and helped challenge early assumptions during theme development.

Finally, the study was conducted within a Swedish healthcare context and focused on a symptom-specific population. Cultural expectations of healthcare professionalism and familiarity with digital services may limit transferability to other settings.

### Future research directions

The findings indicate several directions for future work. First, participants often needed support in making sense of the psychological rationale for their symptoms. Future studies could therefore examine how therapists’ pedagogical work in text form, including clarification and encouragement, helps patients integrate new ways of understanding persistent physical symptoms.

Second, participants described differing preferences for emotional closeness and professional distance. Research is needed to understand how such preferences influence engagement in iCBT and whether matching the therapist’s relational style to the patient’s needs improves the therapeutic process.

Third, participants described strain when tasks felt unclear, overly demanding, or poorly matched to their symptoms. This highlights the importance of identifying common points at which digital programmes may become misaligned with participants’ needs, expectations, or symptom experiences, and of studying how therapists can support participants in navigating task-related or structural difficulties.

Fourth, the structured pacing of weekly modules was experienced as both helpful and constraining. Future work could explore how different levels of flexibility in scheduling and task adaptation influence adherence and motivation.

Finally, the concept of acceptability of the medium for collaboration warrants further refinement. The digital environment became most visible when it hindered progress rather than when it functioned well. Understanding the minimum requirements for a digital medium to support alliance may guide the design of future interventions.

Clinically, the results underline the importance of training therapists in text-based communication, including timely responses, sensitive language matching and clear guidance around challenges in the tasks.

## Conclusions

This study explored how participants in an iCBT programme for NCCP experienced the therapeutic alliance. The findings suggest that alliance in this context was shaped by agreement on goals and tasks, the emotional bond between participant and therapist, and the acceptability of the digital medium for collaboration. Across these dimensions, alliance may be understood as shaped by negotiation processes in which therapist responsiveness helped participants make sense of the treatment rationale, manage strain, and sustain engagement. By broadening the notion of usability into a more comprehensive a*cceptability of the medium for collaboration*, the study extends existing conceptualisations of the digital alliance and highlights the importance of therapist responsiveness in helping participants navigate limitations of digital delivery. Participants’ voices may provide guidance for refining theoretical models that are more context-sensitive, and for shaping clinical training and intervention design in digital psychotherapy.

## Supplementary Information

Supplementary Material 1.

Supplementary Material 2.

## Data Availability

The qualitative interview data supporting the findings of this study are not publicly available due to ethical and confidentiality considerations, as participants did not consent to public data sharing. De-identified data may be made available from the corresponding author upon reasonable request, subject to ethical approval.
