# Supplementary material for: Therapeutic alliance in guided internet-delivered cognitive behavioural therapy: a thematic analysis of patients with non-cardiac chest pain
Source: BMC Psychol. 2026 Jul 15;14:1050. doi: 10.1186/s40359-026-05179-w (PMC13371252; doi:10.1186/s40359-026-05179-w)
Supplement: Supplementary file 2 — Supplementary Material 2. [file 40359_2026_5179_MOESM2_ESM.docx]

**Supplementary File 1: Interview Guide**

**1. Can you tell me about your overall experience of the treatment programme?**

*Optional prompts:*

How did it meet your expectations?

How did it correspond to your goals and needs as you experienced them?

From the beginning, did you believe that the programme could help you?

Did you recognise yourself in the texts and examples?

Did you feel that the programme addressed problems that were relevant to you?

**2. Can you describe how you experienced the content of the different programme modules?**

*Optional prompts:*

Did you feel that you were asked to do things that were relevant and reasonable for you?

Did the content feel helpful in relation to your goals?

Were there parts that felt more or less helpful, or more or less relevant?

**3. Can you tell me about your experience of your therapist? How did you experience your relationship with your therapist?**

*Optional prompts:*

What was important for a functioning collaboration?

How did you experience the therapist’s competence?

How did you experience the therapist’s warmth?

How did you experience the therapist’s authenticity?

Did you feel that you could trust your therapist?

Did you experience your therapist as someone you could confide in?

Did you feel seen and understood by your therapist?

Did you experience a sense of partnership? In what way?

Was there anything in particular that worked especially well?

Is there anything you would have wished the therapist had done differently?

**4. How do you think the therapeutic relationship was affected by the fact that the contact took** place at a distance?

*Optional prompts:*

If you have experience of other forms of treatment, how would you describe the differences?

**5. How was your experience of using the internet platform?**
